# Risk factor profiles and changes in risk factors and cognitive function over 12 months: The CAN‐THUMBS UP Brain Health Support Program Study

**DOI:** 10.1002/alz70860_105618

**Published:** 2025-12-23

**Authors:** Caroline S. Duchaine, Manuel Montero‐Odasso, Haakon B. Nygaard, Paul W.H. Brewster, Diane M. Jacobs, Nicole D. Anderson, Penelope J. Slack, January Durant, Jody‐Lynn Lupo, Howard Chertkow, Howard H. Feldman, Sylvie Belleville, Canadian Consortium on Neurodegeneration in Aging (CCNA), CAN‐THUMBS UP Study Group

**Affiliations:** ^1^ Centre de recherche de l’Institut Universitaire de gériatrie de Montréal (CRIUGM), Montréal, QC, Canada; ^2^ CISSS Chaudière‐Appalaches, Sainte‐Marie, QC, Canada; ^3^ Gait and Brain Laboratory, Canada, ON, Canada; ^4^ Schulich School of Medicine & Dentistry, Division of Geriatric Medicine, Western University, London, ON, Canada; ^5^ University of British Columbia, Vancouver, BC, Canada; ^6^ Cognition & Technology Research Group, Institute on Aging and Lifelong Health, University of Victoria, Victoria, BC, Canada; ^7^ Alzheimer's Disease Cooperative Study (ADCS), University of California, San Diego, La Jolla, CA, USA; ^8^ Rotman Research Institute, Baycrest Academy for Research and Education, Toronto, ON, Canada; ^9^ Alzheimer's Disease Cooperative Study, University of California San Diego, La Jolla, CA, USA; ^10^ University of California San Diego, La Jolla, CA, USA; ^11^ University of Toronto, Toronto, ON, Canada; ^12^ Alzheimer's Disease Cooperative Study, Department of Neurosciences, University of California San Diego, La Jolla, CA, USA; ^13^ Institut Universitaire de Gériatrie de Montréal, Montréal, QC, Canada; ^14^ Department of Psychology, Université de Montréal, Montreal, QC, Canada

## Abstract

**Background:**

Dementia risk factors often coexist and interact in ways that create distinct profiles, which may influence the efficacy of lifestyle‐based interventions aimed at reducing dementia risk. This study aimed to: 1) characterize dementia risk profiles in participants of the CAN‐THUMBS‐UP Brain Health Support Program (BHSP), and 2) evaluate whether these profiles predict responses to a 45‐week educational intervention.

**Method:**

We analyzed data from 296 BHSP participants with complete baseline assessments. Principal component analysis identified risk profiles based on seven modifiable risk factors: physical activity, cognitive engagement, diet, sleep, social and psychological health, vascular health, and vision and hearing. All participants accessed Brain Health PRO, a web‐based educational intervention targeting these modifiable risk factors. Risk factor changes were measured through lifestyle questionnaires administered every three months, and cognitive changes were measured via a neuropsychological test battery at baseline and 12 months. Linear mixed models with repeated measures, adjusted for age, sex, education, and cognitive status evaluated associations between risk profiles and changes in cognition and risk factors.

**Result:**

Three distinct profiles were identified: 1) sleep and social/psychological health, 2) cognitive engagement, and 3) diet, physical activity and vascular heath. Across all profiles, lower baseline scores indicated a higher risk in the factors that characterized each profile. Participants with lower baseline scores in the first two profiles showed greater improvements in associated risk factor domains compared to those with better baseline scores. In the third profile, participants with lower baseline scores showed greater improvements in diet and physical activity, but no change was observed in vascular health. No association was observed between any of the risk profiles and cognitive change. A longer follow‐up or a more intensive intervention beyond online education may be needed to impact vascular health and cognition.

**Conclusion:**

Understanding dementia risk factor profiles and their influence on intervention effects can help inform more personalized prevention strategies. Further research is needed to validate and refine these profiles.

